# Evolutionary models for insertions and deletions in a probabilistic modeling framework

**DOI:** 10.1186/1471-2105-6-63

**Published:** 2005-03-21

**Authors:** Elena Rivas

**Affiliations:** 1 Department of Genetics, Washington University School of Medicine, 4444 Forest Park Blvd., Saint Louis, Missouri 63108 USA

## Abstract

**Background:**

Probabilistic models for sequence comparison (such as hidden Markov models and pair hidden Markov models for proteins and mRNAs, or their context-free grammar counterparts for structural RNAs) often assume a fixed degree of divergence. Ideally we would like these models to be conditional on evolutionary divergence time.

Probabilistic models of substitution events are well established, but there has not been a completely satisfactory theoretical framework for modeling insertion and deletion events.

**Results:**

I have developed a method for extending standard Markov substitution models to include gap characters, and another method for the evolution of state transition probabilities in a probabilistic model. These methods use instantaneous rate matrices in a way that is more general than those used for substitution processes, and are sufficient to provide time-dependent models for standard linear and affine gap penalties, respectively.

Given a probabilistic model, we can make all of its emission probabilities (including gap characters) and all its transition probabilities conditional on a chosen divergence time. To do this, we only need to know the parameters of the model at one particular divergence time instance, as well as the parameters of the model at the two extremes of zero and infinite divergence.

I have implemented these methods in a new generation of the RNA genefinder QRNA (eQRNA).

**Conclusion:**

These methods can be applied to incorporate evolutionary models of insertions and deletions into any hidden Markov model or stochastic context-free grammar, in a pair or profile form, for sequence modeling.

## Background

Probabilistic models are widely used for sequence analysis [1]. Hidden Markov models (HMMs) are a very large class of probabilistic models used for many problems in biological sequence analysis such as sequence homology searches [2-4], sequence alignment [5], or protein genefinding [6-8]. Stochastic context-free grammars (SCFGs) are another class of probabilistic models used for structural RNAs for problems such as RNA homology searches [9-13], RNA structure prediction [14,15], and RNA genefinding [16].

Sequence similarity methods based on HMMs or SCFGs can take the form of profile or pair models and are very important for comparative genomics. These probabilistic methods for sequence comparison assume a certain degree of sequence divergence. For instance, in profile models (either profile HMMs [2-4] or profile SCFGs [12,13]) a sequence is compared to a consensus model. Profile models must allow for the occurrence of insertions and deletions with respect to the consensus, and they do so by using state transition probabilities that assign some position-dependent penalties for modifying the consensus with insertions or deletions. Similarly, in pair probabilistic models [8,16] two related sequences are compared (aligned and/or scored). Pairwise alignments need to allow for substitution, insertion and deletion events between the two related sequences. Substitutions are taken care of by residue emission probabilities, while insertion and deletion events are generally taken care of by state transition probabilities as in the case of profile HMMs.

In the BLAST programs [17], the score of a pairwise alignment is determined using substitution matrices which measure the degree of similarity between two aligned residues. Similarly, in pair probabilistic models, residue emission probabilities are based on substitution matrices. The evolution of substitution matrices has been studied at large for many different kinds of processes: nucleotides, amino acids, codons, or RNA basepairs [18-23]. The evolution of emission probabilities using substitution matrices is easily integrated into probabilistic models both for HMMs [24-29] and for SCFGs [14].

In probabilistic models, insertion and deletion events (indels) are sometimes described by treating indels as an additional residue (gap characters) in a substitution matrix. More often they are described using additional hidden states, where transition probabilities into those states represent the cost of gap initiation and transitions within those states represent the cost of gap extension. If the cost of gap initiation and gap extension are identical, it is referred to as a linear gap cost model. Hidden states allow arbitrary costs for gap initiation and gap extension, which is traditionally referred to as an affine gap cost model. Treating gaps as an extra character in a substitution matrix is equivalent to assuming a linear gap cost model. The parameters that modulate those processes should be allowed to change as the divergence time for the sequences being compared is varied. It has been difficult to combine probabilistic models such as profile and pair HMMs or SCFGs with evolutionary models for insertion and deletions [30-33]. Methods to evolve transition probabilities are not as well developed as those describing substitution matrices, but significant effort is currently aimed at this problem [34-41]. Models incorporating the evolution of insertions and deletions in the context of probabilistic models such as profile HMMs or pair models are a very important goal in order to make those probabilistic models more realistic.

I encountered this problem in working on QRNA, a computational program to identify noncoding RNA genes de novo. QRNA uses probabilistic comparative methods to analyze the pattern of mutation present in a pairwise alignment in order to decide whether the compared nucleic acid sequences are more likely to be protein-coding, structural RNA encoding, or neither. Originally QRNA was parameterized at a fixed divergence time. Motivated by the goal of making QRNA a time dependent parametric family of models, I investigated the possibility of evolving the transition and emission probabilities associated with a given probabilistic model. Since I already had the model parameterized for a given time, I aimed to use that model as a generating point of the whole time-parameterized family of models.

Because QRNA includes both linear and affine gap models in different places, in this paper I propose algorithms to describe the evolution of indels as a (*N *+ 1)-th character in a substitution matrix, and algorithms to describe the evolution of the transition probabilities associated with a probabilistic model.

The purpose of this paper is to describe the general theoretical framework behind these methods. A detailed description of the particular implementation of these algorithms in QRNA and a discussion of the results obtained with "evolutionary QRNA" (eQRNA) will appear in a complementary publication.

## Results

### Evolutionary models for emission probabilities

#### The evolution of emission probabilities without gaps

In order to introduce notation, I will start with a brief review of the current methods for calculating joint probabilities conditional on time, *P*(*i*, *j*|*t*), where *i*, *j *are two residues (for instance, nucleotides, amino acids, RNA basepairs, or codons). *P*(*i*, *j*|*t*) gives us the probability that residues *i *and *j *are observed at a homologous site in a pairwise alignment after a divergence time *t*. Pairwise sequence comparison methods score aligned residue pairs with these joint probabilities either explicitly or implicitly [17]. In explicit generative pair probabilistic models, like the pair-HMMs and pair-SCFG in QRNA, the *P*(*i*, *j*|*t*) terms are referred to as pair emission probabilities.

The evolution of joint probabilities is usually obtained by modeling the corresponding conditional probabilities *P*(*j*|*i*, *t*)as a substitution process in which residue *i *has been substituted by residue *j *over time *t*. Probabilistic models for nucleotide substitutions [18,19,42-44] assume that nucleotide substitution follows a model of evolution that depends on an instantaneous rate matrix,

*Q*_*t *_= *e*^*tR*^,     (1)

where *t *is the divergence time, *R *is the instantaneous rate matrix, and *Q*_*t *_is the substitution matrix of conditional probabilities, that is *Q*_*t*_(*ij*) ≡ *P*(*j*|*i*, *t*). This is a reasonable model used, for instance, to describe nucleotide substitutions in the Jukes-Cantor [42] or Kimura [43] models, or the more general REV model [44]; this is also the evolutionary model used for amino acid substitutions [18,19,21,45,46], codon to codon substitutions [20,47], and RNA basepair to basepair substitutions [14,22,23].

Throughout this paper, I will use the words "divergence time", "divergence", or "time" equivalently to describe the amount of dissimilarity between biological sequences measured as the number of mutations and gaps introduced in the alignment of the sequences. I will never refer to "time" as representing an actual number of years of divergence, since this number cannot be determined intrinsically from sequence data.

Thus, given a rate matrix *R*, *Q*_*t *_(and therefore the desired joint emission probabilities) can be inferred for any desired time using the Taylor expansion for the matrix exponential,



This Taylor series converges in all cases.

There are several ways in which the rate matrix *R *can be determined. One approach is to use analytically inferred rate matrices that depend on a small number of external parameters [42-44,48]. For instance, the HKY model for nucleotide substitutions [48] depends on six parameters: the four stationary nucleotide frequencies, a rate of transitions, and a rate of transversions, which have to be provided externally. Another type of approach uses maximum likelihood methods [21,49,50] in order to estimate a rate matrix numerically from a training set of sequence alignments.

A third approach arises naturally in cases where suitable joint probabilities have already been estimated for a pair model, and we wish to make that model conditional on evolutionary divergence time. This approach starts from the assumption that our point estimate represents sequences at a particular arbitrary divergence time t_*_. For example, a similar assumption was taken to construct the BLOSUM matrices [51], which were obtained as joint probabilities at discrete point estimates from clusters of aligned sequences.

In this third approach the parameters at the generating time t_* _will be used to construct a rate matrix for the process. This approach is motivated by the kind of situation in which we find ourselves with probabilistic methods based on homology such as QRNA: a model has been trained in one kind of data, and the resulting probabilities represent some effective but *fixed *divergence time, and we wish to extend that model to a time-dependent parameterization.

For residue substitution processes, the rate matrix *R *and Q_*_, defined as the substitution matrix at the generating time t_* _[Q_* _≡ *Q*_t_*__], convey exactly the same information. More explicitly, assuming the evolutionary model given in equation (1) we can calculate the rate matrix of the process as a function of Q_* _as



Kishino *et al *[52] introduced the idea of calculating the rate matrix starting from a given substitution matrix using equation (3) and an eigenvalue decomposition of Q_*_. It is worth noting that the matrix equation for the rate *R *can be expressed as a Taylor expansion of the form



which allows for a direct numerical calculation of the rate matrix. The convergence of this series requires only that for every (real or complex) eigenvalue *λ *of matrix Q_*_, then |*λ *- 1| < 1. In addition, for any valid substitution matrix the eigenvalues have to be real and |*λ*| ≤ 1 (see Appendix A). Under these two conditions, the above Taylor series converges so long as the eigenvalues of Q_* _are positive. Therefore the three properties required of Q_* _in order to be able to obtain a rate matrix using the Taylor expansion in equation (4) are that its eigenvalues are all smaller than one (but one that is strictly one), real, and positive. Complex or negative eigenvalues would correspond to oscillatory behaviors, which do not seem to reflect the biology. All the substitution processes I have tested so far for nucleotides, amino acids, and RNA basepairs correspond to real and positive eigenvalues for which the above method is applicable.

**Figure 1:**
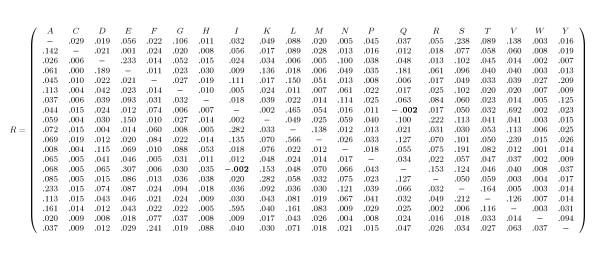
**Rate matrix generated from BLOSUM62. **Rate matrix obtained from the amino-acid substitution matrix BLOSUM62, rescaled to have an average number of one substitution per amino acid. Notice in bold the two off diagonal negative entries.

It is relevant to compare instantaneous rate matrix approaches to the approach used in the PAM amino-acid substitution matrices [53]. The PAM matrices were not generated by calculating a rate matrix, but by estimating from a collection of highly similar sequences the substitution matrix for the time of one substitution per site  ≡ *Q*_*t *= 0.01_, and then calculating *Q*_*t *_at any other (integer t) time by multiplication. This is a discrete approximation that converges to the same answer given by the rate method for very small time units. PAM matrices have been criticized for not being able to capture the substitutions that are observed for more dissimilar sequences. BLOSUM matrices empirically outperform PAM in sequence homology searches, presumably because sequences at larger divergence times were used to calculate the BLOSUM matrices. However, the BLOSUM method is not a time dependent continuous model but a very coarse-grained discretization. There are ways of combining the best of both approaches (more divergent sequence for training and a continuous-time model) to generate rate matrices, for instance by using the resolvent method [54], or using maximum likelihood methods as in the WAG matrices [21]. However, it is also possible to take a discrete BLOSUM matrix, for instance BLOSUM62, and convert it to an underlying rate matrix. The BLOSUM62-generated rate matrix obtained using equation (4) is shown in Figure 1.

A rate matrix can also be derived from the PAM data  by various methods. One exact method is to do an eigenvalue decomposition as presented in [52]. Recently, other methods have been proposed to calculate a rate matrix from the Dayhoff data [55]. These methods still assume that *R *≈ ( - *I*) which corresponds to taking only the first term in the Taylor series for the logarithm in equation (4). This assumption is good only for very closely related sequences. Using the Taylor series allows one to estimate, using the same input data and avoiding the calculation of eigenvalues, the rate matrix to any desired level of precision, independent of the degree of similarity in the training set.

**Figure 2:**
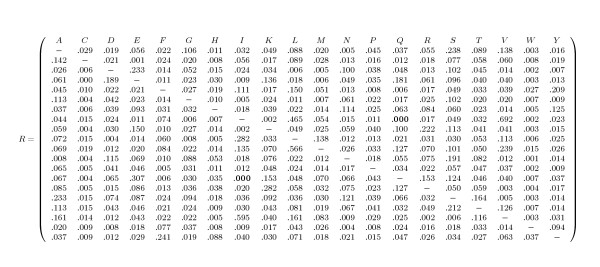
**Regularized rate matrix generated from BLOSUM62 **Regularized rate matrix generated from BLOSUM62 after the QOG algorithm has been applied. The matrix has been rescaled to have an average number of one substitution per amino acid. In this simple case in which there was at most one negative off-diagonal entry per row, the regularization process requires the negative off-diagonal value to be set to zero (represented in bold in this Figure), and to shift the rest of the elements in that row by the corresponding amount so that the sum of all elements is zero. Rows without any off-diagonal negative values remain unchanged from the values obtained in Figure 1.

Notice that the rate matrix obtained using BLOSUM62 (Figure 1) has two off-diagonal negative entries (and if we use more divergent BLOSUM matrices we have more negative off-diagonals). Off-diagonal entries of the rate matrix have to be positive so that *I *+ *δtR *can be interpreted as a substitution matrix for very small times *δt*. This problem is not unique to sequence data. The construction of rate matrices for a Markov process from empirical data using a generating time is also used in mathematical modeling of financial processes such as credit risk modeling [56,57]. In the world of mathematical finances the problem is referred to as the regularization problem. I will use one of following regularization algorithms presented in [57]. The QOG algorithm (quasi-optimization of the generator) regularizes the rate matrix. The QOM algorithm (quasi-optimization of the root matrix) leaves the rate matrix unchanged and regularizes the conditional matrix at a given time if any negative probability appears. Using the QOG algorithm we obtain a regularized version of the rate matrix using BLOSUM62, which is given in Figure 2.

##### Regularization algorithms

Here I reproduce the QOG and QOM regularization algorithms. The proofs for these algorithms can be found in [57]. The QOG algorithm regularizes each row of a rate matrix independently. Given a row in a rate matrix *R*,

*r *= (*r*_1_,...,*r*_*n*_) ≡ (*R*(*i*, 1) ..., *R*(*i*, *n*)),     (5)

the QOG algorithm solves the problem of finding the vector at the minimal Euclidean distance from *r *such that the sum of all its elements is zero, and all elements but one are positive.

The steps of the QOG algorithm are:

1. Permute the row vector so that *r*_1 _= *R*(*i*, *i*).

2. Construct the vector *w*, such that *w*_*i *_= *r*_*i *_- *λ*, where .

3. Obtain the permutation , such that .

4. Construct , for *k *= 2, ..., *n *- 1.

5. Calculate *k*_*min *_= min_*k *_= 2, ..., *n *- 1 {*k *such that *C*_*k *_≤ 0}.

6. Construct the vector



7. The regularized row is given by *r *← *P*^-1 ^(). Finally reverse the permutation of step (1).

The QOM algorithm regularizes each row of a conditional matrix independently. Given a row in a conditional matrix *Q*_*t*_

*r *= (*r*_1_,..., *r*_*n*_) ≡ (*Q*_*t*_(*i*, 1),..., *Q*_*t*_(*i*, *n*)),     (6)

the QOM algorithm solves the problem of finding the vector at the minimal Euclidean distance from *r *such that the sum of all its elements is one, and all elements are positive.

The steps of the QOM algorithm are:

1. Construct the vector *w*, such that *w*_*i *_= *r*_*i *_- *λ*, where .

2. If all *w*_*i *_are non negative, *r *← *w *is the new regularized row.

3. Otherwise, obtain the permutation , such that .

4. Construct , for *k *= 1,..., *n*.

5. Calculate *k*_*max *_= max_*k *= 1,..., *n *_{*k *such that *C*_*k *_≤ 1}.

6. Construct the vector



7. The regularized row is given by *r *← *P*^-1 ^().

##### A 4 × 4 example starting from joint probabilities at a given generating time

As an review of these techniques, I will use a set of 4 × 4 single-nucleotide joint probabilities *P*(*i*, *j*|t_*_) for *i*, *j *= {*a*, *c*, *g*, *t*} at a particular generating time t_* _to construct the corresponding rate matrix.

In this example, the joint probabilities at the generating time using the matrix notation P_*_(*ij*) ≡ *P*(*i*, *j*|t_*_) are given by,



These 4 × 4 pair-nucleotide probabilities are taken from the program QRNA. They were calculated according to [16] by marginalizing codon-codon joint probabilities which were constructed from the BLOSUM62 matrix of amino acid substitutions. These 4 × 4 probabilities can be viewed as a particular example of the REV model [44]. Note that the sum of all elements of P_* _adds up to one, and the matrix is symmetric.

The marginal probabilities defined as *p*_*i *_= ∑_*j *_*P*(*i*, *j*|t_*_) can be calculated from the joint probabilities to be,

*p *= (*p*_*a*_, *p*_*c*_, *p*_*g*_, *p*_*t*_) = (0.2836, 0.2311, 0.2531, 0.2322).     (8)

Similarly, the conditional probabilities *P*(*j*|*i*, t_*_) can be calculated from the previous joint and marginal probabilities using the relationship *P*(*i*, *j*|t_*_) = *P*(*j*|*i*, t_*_) *p*_*i*_. Using the matrix representation Q_*_(*ij*) ≡ *P*(*j*|*i*, t_*_) we have,



Notice how the sum of the elements in each row adds up to one. Notice also how Q_* _is quite different from the identity matrix, which means that we have started with a quite divergent generating time.

If we assume a homogeneous Markov substitution process, we can interpret the conditional probabilities Q_* _as the matrix of substitution probabilities at the generating time. Thus, we can characterize the underlying evolutionary process by its instantaneous rate of evolution, which can be calculated from Q_* _using equation (4). The resulting rate matrix *R *(up to an arbitrary scaling factor t_*_) is given by,



This rate matrix has all the good properties: (*i*)"Normalization": the sum of the elements of each row is zero. (*ii*) "Reversibility": *p*_*i *_*R*_*ij *_= *p*_*j *_*R*_*ji*_. The process is reversible by construction because we started with symmetric joint probabilities. (*iii*) "Saturation". The rate matrix converges at time infinity to the given marginal probabilities in equation (8). We can test saturation by using equation (2) and calculating the substitution matrix for a very large time. For instance, for *t *= 10t_* _we have



Saturation (or stationarity) of a Markov process is a necessary consequence of (*i*) normalization and (*ii*) reversibility. Appendix A shows a derivation of the previous statement which was useful for me (and hopefully for some readers) when studying the behavior at *t *= ∞ of more complicated evolutionary models. Therefore, starting from joint probabilities as in this example, we can always interpret the marginal probabilities as the stationary probabilities of the evolutionary process.

In summary, starting with a single set of joint probabilities at one particular generating divergence time t_*_, we calculate the joint probabilities at any other arbitrary time, assuming an exponential model of evolution. To that effect, given the particular set of joint probabilities (7) we have calculated the corresponding rate matrix (10) by Taylor expansion. Thus we can estimate the substitution matrix/conditional probabilities at any other arbitrary time, simply using equation (2), and reconstruct the joint probabilities at any other arbitrary time. For instance, for *t *= 0.3t_* _we obtain,



This method allows us to evolve pair emission probabilities corresponding to different processes (in addition to the 4 × 4 nucleotide emissions) for instance 20 × 20 amino acid-to-amino acid joint emission probabilities, 64 × 64 codon-to-codon joint emission probabilities, or 16 × 16 RNA basepair-to-basepair joint emission probabilities. Thus, this method is useful to be applied in combination with pair HMMs or pair SCFGs already parameterized at one fixed divergence time to make their emission probabilities a time-dependent family.

#### The evolution of emission probabilities with indels treated as an extra character

Substitution processes (even if describing multi-nucleotide events such as codon evolution or RNA basepair evolution) are not enough to describe the full evolutionary relationship between two biological sequences. We also need to consider indels, for which we need to introduce more complicated models of evolution than the one described so far.

Indels have traditionally been a problem for phylogenetic methods. Programs to construct phylogenetic trees from data such as PHYLIP [58], PAUP^* ^[59], and other phylogeny packages [60-64] treat gaps as missing data. The theoretical description of the evolution of gaps in a probabilistic fashion reached a landmark with the Thorne/Kishino/Felsenstein (TKF) model [30,31]. The TKF model however is hard to implement in combination with a probabilistic model such as an HMM, although an active area of research exists in that direction [36,39,40]. A more direct attack to the problem of introducing phylogeny into existing probabilistic models originated with the concept of tree HMMs [34,35]. The tree HMM method models the evolution of the parsing of different sequences through an HMM. This approach is more related with the evolution of transition probabilities, and I will discuss it later on in this paper.

Here I am going to describe a method for the evolution of indels under the assumption that they behave like an additional residue added to a *N *× *N *residue substitution matrix. This is a simplification of the problem because it forces indels to have linear penalties (that is, the cost of opening an indel in an alignment or the cost of extending it with one more indel character is the same) and to behave independently of each other (that is, successive indel characters in one sequence will be treated as independent events, rather than as a single indel of *n *residues long). Despite its apparent simplicity, this approach poses interesting problems in parameterizing evolution.

Let us review some of the implications of insertion and deletion processes. The treatment that pair models give to pairwise alignments can be interpreted (if we assume reversibility, as is the case here) with all generality as if one of the sequences is the ancestor of the other one. For any two aligned residues we assume that they can be related by a substitution process. For a residue aligned to a gap we assume that either a residue in the ancestor was deleted in the descendent sequence, or that a residue not present in the ancestor appeared in the descendent sequence.

An stochastic insertion–deletion process also involves insertions followed by subsequent deletions. These events leave no trace in pairwise alignments because alignments usually do not retain gaps aligned to gaps. However, when we are treating indels as an extra character, we have to account for such events.

If we were given ideal alignments with all their gap-to-gap aligned columns we could estimate from data the (*N *+ 1) × (*N *+ 1) extended joint probabilities at a generating time, . Because that is not the case, we need to make some inference about . Let us represent with Δ, such that 0 ≤ Δ ≤ 1/2, the expected frequency of observed gaps with respect to the total number of residues in pairwise alignments at a particular time t_*_. The parameter Δ, can be estimated from data, or it could be estimated according to the TKF model [30] as



if we knew the values for the rate of insertions *λ *and the rate of deletions *μ*, such that 0 <*λ *<*μ*.

Let us represent with Δ' the expected frequency of missing gap-to-gap aligned columns in a pairwise alignment at a particular time t_*_. One can estimate Δ' as the expected length of insertions that were later deleted without leaving any trace in current sequences. The probability of a stretch of *l *gap-to-gap characters is given by the geometric distribution density *ρ*(*l*) = (1 - Δ^2^) Δ^2*l*^. Therefore Δ' is given by,



Using these two parameters and the joint probabilities in the absence of gaps at the generating time P_*_() we can construct the set of (*N *+ 1) × (*N *+ 1) extended joint probabilities at t_* _as



where we have assumed independence for the joint probability of a residue and a gap. The normalization factor Ω = 1/(1 + 2Δ + Δ') represents the fact that the observed Δ is different from the value we would have obtained had we known the complete alignment.

Another implication of insertion and deletions appears in the behavior of the marginal probabilities of single residues and indels. At *t *= 0 when sequences have not yet diverged, the marginal probability of finding a gap in an alignment should be zero. In the limit *t *= ∞, the pairwise alignment of two finite-length sequences is going to be dominated by gap-to-gap alignments, which implies that as the divergence time increases the marginal probability of a residue becomes negligible, while the marginal probability of a gap becomes one in the limit *t *= ∞. Our evolutionary model has to be able to accommodate such saturation frequencies.

##### A step-by-step description of the algorithm for the evolution of gaps as an extra residue

I will start by describing the steps to implement the method before explaining how to derive those steps. This method can be applied starting from two different situations: starting from a *N *× *N *set of joint probabilities at a generating time that need to be extended to allow indel characters and evolved with time; or starting from a given *N *× *N *rate matrix that needs to be extended to allow indel characters.

Suppose we start with a *N *× *N *set of joint probabilities P_* _at a generating time t_*_, where *p *stands for the marginal probabilities and Q_* _represents the set of conditional probabilities associated with P_*_.

1. Extend the joint probabilities at the generating time t_* _to a (*N *+ 1) × (*N *+ 1) matrix of joint probabilities  of the form,



where Δ is a parameter which represents the expected frequency of gaps with respect to the total number of residues in an pairwise alignment at t_*_, and which satisfies the condition 0 ≤ Δ ≤ 1/2. The parameter Δ' is given in terms of Δ as , and the normalization constant is given by Ω = 1/(1 + 2Δ + Δ'). The indices with hats () stand for the *N *residues, and exclude the gap character, which I represent with the symbol -.

The (*N *+ 1) × (*N *+ 1) extended conditional probabilities at the generating time  are given by,



2. Construct the (*N *+ 1) × (*N *+ 1) extended rate matrix *R*^*ε *^as



where



3. Calculate the exponential of the rate matrix  using the Taylor expansion,



4. Construct the extended matrix of conditional probabilities at arbitrary time  as



where the matrix *Q*_0 _is given by



where  are the original marginal probabilities of P_*_, and the probability 0 <*q*_0 _≤ 1 is given by . The function *δ*(*ij*) is a Kronecker delta which takes value one for *i *= *j *and zero otherwise. The case *q*_0 _= 1 corresponds to the extreme case in which the *N *+ 1 gap residue does not evolve.

5. Construct the extended marginal probabilities  as



where the probability of a gap at time *t *is given by,



I call this process "quasi-stationary" because the background frequencies  () at any finite time are always proportional to the original *N*-dimensional background frequencies . This result is a concequence of the fact that the first *N *elements of the last row of *Q*_0 _are proportional to the *N *stationary frequencies . On the other hand, while remaining "quasi-stationary" the background frequencies evolve from (, 0) at time zero towards "all gaps" at time infinity, *i.e. *lim_*t *→ ∞ _Λ_*t *_= 1. This behaviour at time infinity is the consequence of the particular value of *q*_0 _selected in the previous step.

6. Finally, construct the evolved (*N *+ 1) × (*N *+ 1) joint probabilities at arbitrary time  as



The expression for Λ_*t *_in equation (24) guarantees reversibility, that is, that the extended  constructed according to the above expression are symmetric.

For the other starting situation, in which we have a *N *× *N *rate marix *R*, the procedure to generate a (*N *+ 1) × (*N *+ 1) quasi-stationary reversible evolutionary model is the following:

1. Construct the (*N *+ 1) × (*N *+ 1) extended rate matrix *R*^*ε *^as



where we have extended the *N *× *N *rate matrix *R *with the parameter *β *> 0.

The instantaneous rate is given by,



Thus *β *is the instantaneous rate of deletion of a character, while -*β*(1 - *q*_0_) is the rate of insertion of character . (More complicated models in which the rate of deletion is different for different characters are also possible.) Notice that *q*_0 _= 1 corresponds to the case in which the rate of insertions is zero.

2. Find  analytically, if an analytic expression for *R*^*ε *^is given by solving the differential equation *d *() / *dt *= *R*^*ε *^, or numerically, proceeding as in step (3) of the previous procedure.

3. Proceed as in steps (4)-(6) of the previous procedure.

##### A 5 × 5 example starting from joint probabilities at a given generating time

We start with the generating joint probabilities P_* _in the 4 × 4 example in equation (7), which we want to extend to a 5 × 5 matrix by adding a gap character. For this example, I have selected the arbitrary value for the gap parameter Δ = 0.18.

The 4 × 4 joint probabilities in equation (7) augmented to a 5 × 5 matrix  using the gap parameter Δ = 0.18 (which implies that Δ' = Δ^2^/(1 - Δ^2^) = 0.0335) is given by,



The conditional probabilities  (*ij*) = *P*^*ε *^(*j*|*i*, t_*_) are given by,



The extended marginal probabilities at the particular time instance t_* _are given by,

 = (*p*_*a*_, *p*_*c*_, *p*_*g*_, *p*_*t*_, *p*_-_) = (0.2402, 0.1957, 0.2143, 0.1966, 0.1532),     (30)

which are quasi-stationary with respect to the 4 × 4 stationary probabilities *p *= (0.2836, 0.2311, 0.2532, 0.2322) we started with in equation (8).

The matrix of conditional probabilities at time zero using expression (22) is given by,



The rate matrix for this example, calculated using the Taylor expansion described in equation (18) takes the value,



One should not be concerned to see a whole row of zeros for this rate matrix. For this generalized model the instantaneous rate of evolution is not directly given by the rate matrix; instead, the instantaneous rate of evolution is given by,



In this example, the instantaneous rate of evolution takes the form,



One should not be concerned either by having some negative off diagonal components. For small times *δt*, the conditional matrix is given by,



Therefore, in order to have a proper matrix of conditional probabilities for sufficiently small *δt*, it is necessary to satisfy the following condition for each pair of indices *i*, *j*,

if *Q*_0_(*ij*) = 0 then (*Q*_0_*R*^*ε*^)(*ij*) > 0.     (36)

In this case, the off-diagonal components of the last row of *Q*_0 _are non-zero, which allows us to have negative off-diagonal elements for that row in the instantaneous rate matrix *Q*_0_*R*^*ε*^.

With the 5 × 5 rate matrix in hand, we can apply steps (3) and (4) to obtain the conditional probabilities at any arbitrary time . For instance for *t *= 0.3t_* _we obtain the following evolved conditional probabilities:



The quasi-stationary marginal probabilities are constructed using the result  = 0.0487, and the 4 × 4 stationary probabilities *p *= (0.2836, 0.2311, 0.2532, 0.2322), following step (5) of the algorithm as,



Finally, using equation (25), for *t *= 0.3t_*_, we obtain the following evolved joint probabilities



Notice that this matrix is symmetric, which is the result of having imposed reversibility for any arbitrary divergence time.

We can also see by calculating the conditional probabilities at large divergence times how these probabilities evolve towards their saturation values given by (0, 0, 0, 0, 1). For instance, for *t *= 30t_* _we have,



##### An example starting from a rate matrix: The Jukes-Cantor model extended to gaps

As an example of a situation in which we start with a rate matrix, let us consider the generalization of the Jukes-Cantor model [42] to a 5 × 5 evolutionary model with a gap character. The original Jukes-Cantor model assumes that all nucleotides mutate at the same rate *α *> 0 which is represented by the rate matrix



In this simple case the conditional matrix *Q*_*t *_= *e*^*tR *^can be found analytically by solving the matrix differential equation . Because of the symmetries of the problem we can write



with the condition *r*_*t *_+ 3*s*_*t *_= 1. We then obtain the following differential equations





and the solutions are,





By taking the limit *t *= ∞ in the previous two equations, one can see that the saturation frequencies of the Jukes-Cantor model are *p*_*i *_= 0.25 for *i *= *a*, *c*, *g*, *t*.

The 5 × 5 extended Jukes-Cantor rate matrix *R*^*ε *^is constructed by adding a rate of mutation to a gap represented by the quantity *β *≥ 0 which in principle we will assume is different from the rate of substitutions *α*,



We also introduce the matrix at time zero *Q*_0 _which depends on the probability parameter 1 ≥ *q*_0 _> 0,



where the particular case *q*_0 _= 1 is only allowed if simultaneously *β *= 0, and corresponds to a trivial extension of the original Jukes-Cantor model in which the gap character does not evolve.

The conditional matrix at arbitrary time is given by  = *Q*_0_. The symmetries of the problem in this case allow us to parameterize  as



with the conditions *r*_*t *_+ 3*s*_*t *_+ *γ*_*t *_= 1 and 4*ξ*_*t *_+ *σ*_*t *_= 1.

Introducing the matrix *M*_*t *_≡ , we can parameterize



which implies that



*σ*_*t *_= (1 - *q*_0_) γ_*t *_+ *q*_0_.     (52)

The differential equation to calculate *M*_*t *_takes the form  = *R*^*ε *^*M*_*t*_, which translates into the differential equations,







Which are satisfied by





*γ*_*t *_= 1 - *e*^-*βt*^.     (58)

And in addition we have



*σ*_*t *_= 1 - (1 - *q*_0_) *e*^-*βt*^.     (60)

In the limit case *β *= 0, the solutions for *r*_*t *_and *s*_*t *_reduce to those of the original Jukes-Cantor model with the trivial additions of *σ*_*t *_= 1, *ξ*_*t *_= 0 and *γ*_*t *_= 0, after setting *q*_0 _= 1.

The extended Jukes-Cantor model depends on three parameters: the rate of nucleotide substitution *α *> 0, the rate of nucleotide deletion *β *≥ 0, and the parameter 1 ≥ *q*_0 _> 0. What is the meaning of *q*_0 _? *q*_0 _controls the saturation frequencies (*i.e*. the background frequencies at time infinity), as well as the background frequencies at any other finite time. For *β *> 0 and 1 >*q*_0 _> 0, taking the limit *t *= ∞ in equations (56)-(60), one can see that the saturation probabilities are given by (0, 0, 0, 0, 1). At any other finite time, the background frequencies of the model are quasi-stationary with respect to the background frequencies of the original Jukes-Cantor model, and are given by



*p*_*t *_(-) = Λ_*t*_.     (62)

Imposing the reversibility condition  in particular we obtain (1 - Λ_*t*_)*γ*_*t *_+ Λ_*t*_*σ*_*t *_= Λ_*t *_which implies,



Therefore *q*_0 _controls how fast the background frequencies approach the saturation probabilities (0, 0, 0, 0, 1) through the factor Λ_*t*_. For a given *β*, the larger *q*_0_, the faster Λ_*t *_approaches one. (Note that Λ_*t *_always approaches one as *t *goes to infinity.)

At first glance, it looks like *q*_0 _could take any value including one in the solution for the extended Jukes-Cantor model. *q*_0 _= 1 would result in fixed background frequencies of the form (0, 0, 0, 0, 1), which is an undesirable result, and the value *q*_0 _= 1 would have to be excluded when *β *> 0. In fact, the limit to the ungapped Jukes-Cantor model has to be taken by setting *β *= 0 first, and then *q*_0 _= 1. In that way, Λ_*t *_= 0 for all times, which is the correct result for the original Jukes-Cantor model.

##### Derivation of the algorithm for a (N + 1) × (N + 1) quasi-stationary and reversible evolutionary process

Unlike the ungapped *N *× *N *case in which the marginal probabilities are time independent, in the presence of gaps the marginal probabilities have to evolve with time. In fact, as I discussed earlier, the marginal probability of a gap  (-) has to evolve from zero at time zero to one at time infinity. As a result of that observation, probabilistic evolutionary models with *Q*_0 _≠ *I *are necessary in the presence of gaps in order to maintain reversibility. The reason for this requirement is the following: for an evolutionary model of the form *e*^*tR*^, reversibility implies that there is some p_* _such that p_*_Q_* _= p_* _[see Appendix B, equation (202)]; it follows then that p_*_R = 0, and therefore p_*_e^*tR *^= p_* _for arbitrary time *t*. Thus, under a reversible model of the form *Q*_*t *_= *e*^*tR*^, marginal probabilities do not evolve with time. On the other hand, if *Q*_0 _≠ *I *then the condition p_*_Q_* _= p_* _does not imply p_*_R = 0 for the rate matrix *R*, and therefore it does not impose p_* _as the marginal probabilities for arbitrary *t*. (See appendix B for more details on this point.)

Therefore, to model the evolution of gaps we need to generalize the evolutionary model to have the following form

 = *Q*_0_.     (64)

The matrix *Q*_0 _can be parameterized in following form,



This matrix depends on one additional parameter *q*_0_. The particular dependency *Q*_0_(-, ) ∝ , is necessary to obtain quasi-stationary reversibility of the marginal probablilities.

The rate matrix is now a function of *Q*_0 _and , and takes the form



where the matrix  has the form,



where



Notice that *Q*_0 _may be inverted as long as 0 <*q*_0 _≤ 1.

With respect to the marginal probabilities we have that at the generating time t_* _because of the way the extended probabilities P_* _were constructed we imposed a quasi-stationary behavior of the form,



where



The generalized conditional matrix in (64) also saturates at very large times, and the saturation probabilities (*i.e*. the marginal probabilities at infinity) are given by those of the rate matrix, that is lim_*t*→∞ _ = lim_*t*→∞ _ (see Corollary A.1). Because of the relationship in equation (66) between the rate matrix and the matrix , the saturation probabilities  are given by the condition (see Appendix B),



Then using equation (71) we can see that the saturation probabilities maintain the quasi-stationary property that was imposed at the time instance t_*_, and are given by,



where



As I discussed before, it is reasonable to impose that at infinity all we find is gaps, *i.e *Λ_∞ _= 1.0, which implies *η *= 1 and



Notice that because the relationship given in equation (14) between Δ' and Δ, then 0 <*q*_0 _< 1.

For an arbitrary time we have the reversibility relationship



This equation is satisfied by construction in the *N *× *N *subspace. By inspecting the implications of the above equation for the gap index, we obtain an expression of Λ_*t *_(the marginal probability of a gap) at arbitrary time that allows us to have quasi-stationary reversible evolution. The function Λ_*t *_is given by,



### Evolutionary model for transition probabilities

**Figure 3:**
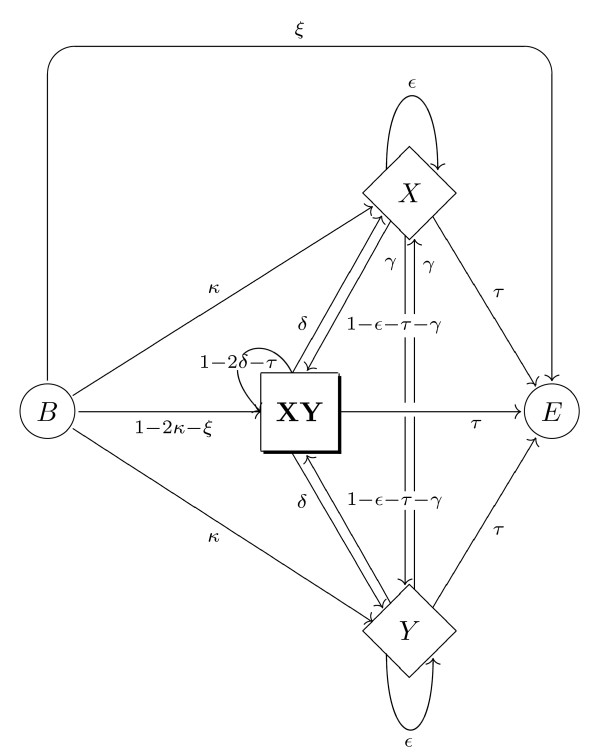
**A pair-HMM model. **Description of a pair-HMM model. The three states: Emit-a-pair (XY), Emit-X (X), and Emit-Y (Y) have four possible transitions each, which we are going to make time-dependent functions. This a geometric model, in which the expected length is given by 1/*τ*. In order to generate alignments with the same expected length at all times, we will leave the parameter *τ *(the transition of each of the three states into the exit state) unchanged with time. The figure shows the transition probabilities having the following properties: 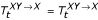, 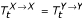, 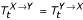, 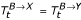. These properties guarantee that the model is reversible.

The standard way in which comparative probabilistic models allow for insertions and deletions is by introducing several additional states with their corresponding transition probabilities. For instance, in a pair-HMM for sequence alignment (Figure 3) the presence of gaps requires the introduction of two states ("X" and "Y") which emit a nucleotide in only one of the two sequences. The probabilities associated with transitioning in and out of those states control the "gappiness" of the alignment. Therefore the evolution of these parameters with time is necessary in order to model different degrees of sequence divergence.

There has been a continued effort on improving the accuracy of the evolution of emission probabilities (*i.e*. substitution matrices) such as allowing correlations between the rates at different sites [65,66], improvements in the derivation of rate matrices from sequence data [23,67], or estimating multiple nucleotide changes [68]. In comparison, the ideas to describe the evolution of transition parameters in probabilistic models are much less standardized [34-40].

The goal of this section is to describe the evolution of transition probabilities. For instance, in the pair-HMM of Figure 3 the transition probabilities from the **"XY" **state to the "X" or "Y" states describe the introduction of gaps in one of the two aligned sequences, using an affine penalty. These transitions should be zero when the sequences have not yet diverged (time zero), but they should be maximal at infinite divergence. In between these two extremes, it is desirable to model the transition probabilities changing with divergence time. These methods are termed "evolutionary" because the transition probabilities will be parameterized with time, using functions that are generalizations of the Markov process that probabilistic evolutionary models assume for substitutions. Unlike the TKF model [30,31] and other related evolutionary models [32,33,41], the approach presented here will not describe the actual underlying evolutionary process that may have generated one sequence from another.

The tree-HMM method [34,35,37] is possibly the method closest to what I develop here. A tree HMM tries to model the phylogenetic relationship between related sequences by modeling the parsings of different sequences through the model. In a tree HMM it is not the actual transition probabilities of the HMM, but the parsing of the different sequences through the models that are evolved using rate matrices that resemble the diagonal rate matrices introduced in the first of the methods described below. Here I want to generate pair or profile probabilistic models that when comparing two related sequences are able to accommodate to the degree of divergence observed between the two sequences, and I intend to do that in a continuous-in-time and probabilistic fashion, using the smallest possible number of free parameters. No evolutionary history of individual insertion/deletion events will be generated; only *a posteriori *would an evolutionary history be established by comparing sequences (in the case of a profile model) or alignments (in the case of pair models) generated by the model at different times.

I present two methods to evolve transition probabilities. One of the methods considers the evolution of a vector of transition probabilities. In this method, the value of the transition probabilities at time zero and time infinity are input parameters, which gives a relatively large number of free parameters. In the second, more restrictive, method the transitions associated with several states are assumed to evolve under the same evolutionary process. This condition constrains some of the free parameters, but does not fix them all completely. When the more restrictive conditions are used, both algorithms give the same results. These two algorithms are applicable to most pair and profile probabilistic models, be they HMMs or SCFGs, generalized or not. I present an example of the evolution of a vector probability vector for a pair HMM, and an example of the evolution of a matrix of transition substitutions for a profile HMM.

#### Evolution of a vector of transition probabilities

##### A step-by-step description of the algorithm

Let us start by providing the recipe to apply the algorithm:

1. Given a transition probability vector



2. Assume its set of values is known at the three particular time instances of *t *= 0, *t *= t_*_, and *t *= ∞, named *q*_0_, q_*_, and *q*_∞_. Assume each component *i *in these probability vectors satisfies one of the following three conditions,

*q*_0_(*i*) <q_*_(*i*) <*q*_∞_(*i*) or *q*_0_(*i*) >q_*_(*i*) >*q*_∞_(*i*) or *q*_0_(*i*) = q_*_(*i*) = *q*_∞_(*i*), for all *i*.     (78)

3. If the three input vectors satisfy the condition,



where *r *> 0 is a real number independent of *i*, then calculate *q*_*t *_at an arbitrary time *t *(0 <*t *<*t*_∞_) as



Normalization of the vector *q*_*t *_is guaranteed by equation (79).

4. Otherwise *q*_*t *_is given by the following expression



where the function *w*_*t *_is given by



##### An example: evolution of the transition probabilities of a pair-HMM "XY" state

Consider the transition probability vector associated to the "XY" state of the pair-HMM given in Figure 3,



which describe the four possible transitions from a correlated emission of two nucleotides to another correlated emission in both sequences (); to a gap in sequence Y (); to a gap in sequence X (); or to end the alignment ().

Below are some arbitrary values for the transition vector at divergence times: *t *= 0, *t *= t_*_, and *t *= ∞ associated with state "XY", *q*^*XY*^,



The transition *T*^*XY*→*E *^= *τ *is related to the expected length of the alignments generated using the model. We typically want to keep that transition invariant through time, and correlated with the alignment length L: *τ *= 1/*L*. (This pair HMM produces sequences with a geometric length distribution of mean 1/*τ*.) The other three transitions change with time from a situation of no gaps at time zero, to a situation at time infinity in which all there is present is gaps, because no residue in either sequence has a homologous residue in the other.

Transition probabilities at *t *= 0 and *t *= ∞ can be stated from first principles. Transitions at the generating time t_*_, are estimated from data, at the same time that emission probabilities are estimated. The transition probabilities at any other time are given by applying the algorithm. Using equation (81) we obtain,



Similarly to this "XY" state case, all the other transition probabilities that appear in the pair model of Figure 3 could be continuously parameterized with the divergence time of the alignment being scored. This algorithm can be applied to any full set of transition probabilities emerging from a particular state in a given probabilistic model that must evolve with time.

##### Connection with a tree-HMM 2×2 match-transition matrix

In the original representation of a tree-HMM [35] the idea of a match-transition matrix is introduced. If one parse through the HMM generated a Match to Match (*MM*) transition, while another parse through the model generated a Match-to-Delete (*MD*) transition, one can consider the substitution of *MM *by *MD *similarly to a substitution of residues by the conditional probability *P*(*MD*|*MM*, *t*). This leads to the concept of a 2 × 2 match-transition matrix given by,



which in [35] is parameterized with two real numbers *r *≥ 0, and 0 ≤ *a *≤ 1 as



Tree-HMMs model the evolution of paths though the HMM. In contrast, the method proposed here models the evolution of the transition probabilities of the model themselves. However, one can see that the match-transition matrix is closely related in form to the model we have proposed here. Introduce the probability vectors,



For *t *= 0 and *t *= ∞ they have the following values,



It is easy to see that the match-transition matrix given in equation (87) can be rewritten as,





for a diagonal rate matrix . Such diagonal rate matrix does not require additional normalization because it corresponds to the case described in equation (80).

##### Derivation of the algorithm to evolve a vector of transition probabilities

To describe the evolution of transition probabilities, the simple exponential models used for substitution matrices are not sufficient. I propose to adopt a generalization of the exponential model of the form,



where *I *is the *n *× *n *identity matrix, *r *is a vector still to be identify, and a *R *is a *n *× *n *rate matrix.

This model simply adds to the exponential term a time independent vector *a*,



Because *q*_*t*=0 _= *q*_0_, then it is necessary that *a *= *q*_0 _- *r*, thus giving the expression in equation (92). Note that this is the most general solution of a differential equation of the form  ∝ (*q*_*t *_- *a*). Until now it was always assumed that the constant term was zero, that is *r *= *q*_0_. The freedom added by including a term constant in time is that, while before the behavior at *t *= ∞ was solely controlled by *e*^*tR*^, now the additional term also contributes to that limit.

An immediate consequence of this generalization is that the rate matrix is not now sufficient to determine the whole evolutionary process. In addition to the rate matrix, the probability vector must also be specified at time zero (no divergence) and at time infinity (all mutations have saturated) such that,



The exponential of the rate matrix *R *has the general form,



for some real eigenvalues . If conditions are restricted to the case in which *k*_*i *_> 0, ∀*i*, the immediate consequence of working with positive eigenvalues is that,



There is then a simple relationship between the vector *r *and the values of the probabilities at time zero and saturation,

*q*_∞ _= *q*_0 _- *r*.     (97)

Therefore, we can write with all generality



However, for the given information (*q*_0_, q_*_, *q*_∞_), the time-parameterized vector *q*_*t *_in (98) is still underdetermined.

In order to reduce the amount of freedom, I assume that *e*^*tR *^is diagonal (*i.e. U *= *I*). Diagonal rate matrices have been used in other contexts of generalized evolution such as the tree-HMM model [34,35]. Then we have,



At this time the known probabilities at the generating time t_*_, q_*_, have not yet been used. These are,



Thus we obtain



which can be solved for *k*_*i*_,



The condition *k*_*i *_> 0 translates into 0 < < 1, or



This condition has two solutions:

*q*_0_(*i*) <q_*_(*i*) <*q*_∞ _(*i*) or *q*_0 _(*i*) >q_* _(*i*) >*q*_∞ _(*i*).     (104)

Even though this model was derived under the conditions of equation (104), it also extends to the degenerate case where for some *i *we have

*q*_0_(*i*) = q_*_(*i*) = *q*_∞_(*i*),     (105)

since this simply corresponds to these parameters undergoing no evolution at all.

Therefore if the input column vectors satisfy one of the three previous conditions for each one of their elements, the parametric expression is



A normalization condition has not yet been imposed. Using the unity vector *u*^*T *^= (1,..., 1), normalization requires that



For an evolutionary model of the form *q*_*t *_∝ *e*^*tR *^normalization requires that *e*^*tR*^*u *= *u *for arbitrary times. I refer to this property as the strong normalization condition. The normalization of a generalized evolutionary model of the form  requires the weaker condition (*q*_0 _- *q*_∞_)^*T*^*e*^*tR*^*u *= 0. This property is always true for a rate matrix that satisfies the strong normalization condition. I refer to this property as the weak normalization condition.

In order to obtain the strong normalization condition automatically it is necessary to have a rate matrix of the form *R *= *U diag *(0, - *λ*_2_,..., -*λ*_*n*_) *U*^-1 ^(see Appendix A, equation (193)). Such a type of rate matrix is not appropriate to describe the evolution of a probability vector, since such rate matrix cannot be uniquely inferred from the three input probability vectors *q*_0_, q_*_, *q*_∞_. For that reason, I have explored the use of rate matrices of the diagonal form *R *= *diag *(-*k*_1_,..., -*k*_*n*_), which can be inferred from the three input probability vectors *q*_0_, q_*_, *q*_∞ _using expression (102). Such diagonal rate matrices, however, in general do not satisfy the strong normalization condition, thus the weak normalization normalization condition must be obtained by other means.

Define *w*_*t*_:



If the input vectors satisfy the condition for all *i*,



for some real number *r *> 0, then the rate matrix has the particular form *R *= *diag*(-*r*,..., -r), the function *w*_*t *_= 0 for arbitrary times, and the weak normalization condition is satisfied automatically.

The previous condition is in general too restrictive. If the previous condition is not satisfied, by construction *w*_*t *_is zero at *t *= 0, *t *= t_* _and *t *= *t*_∞_, but in general *w*_*t *_≠ 0 for *n *> 2. Normalization is then achieved (107) by modifying our definition of *q*_*t *_to



The final expression is



#### Evolution of a matrix of transition probabilities

**Figure 4:**
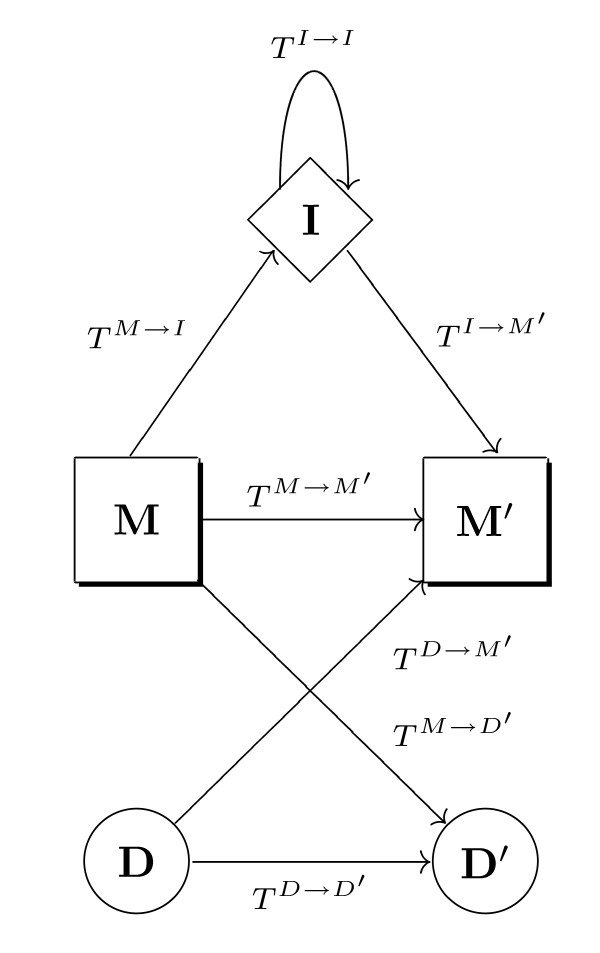
**Part of a profile HMM model. **For a profile HMM we depict the transition probabilities associated with the states of a given consensus position in the profile: Match (*M*), Insert (*I*), and Delete (*D*). The three states corresponding to the next position in the profile are referred to with primes. The Match state has three transitions (into *I*, *M'*, and *D'*), while the Insert and Delete states have two transitions each (into *I *and *M'*, and into *D' *and *M' *respectively).

In some cases, several states of a model correspond to a particular evolutionary event, and it seems natural to expect that their transitions would evolve under the control of the same rate matrix. For instance, in a profile HMM (Figure 4) I will consider the joint evolution of the transitions of three states associated with a given consensus position: Match (*M*), Insert (*I*), and Delete (*D*).

For a collection of *m *states *S *= {*S*_1_,..., *S*_*m*_} that transition into a collection of *n *states *E *= {*E*_1_,..., *E*_*n*_}, consider the set of all transition probabilities emerging from the *m *originating states *S *and ending in the *n *states *E*,



The set of *S *and *E *states do not have to be mutually exclusive, and some *E *states can also be part of the *S *set. The set of *E *states also has to be complete, in the sense that



On the other hand, not all *E *states need to be reached by a given  state; some transitions may be forbidden by design. For instance, for the states associated with a consensus position in a profile HMM the set of originating states is *S *= {*M*, *D*, *I*}, and the set of ending states is *E *= {*M'*, *D'*, *I*}, where the prime index indicates the next position in the profile. The condition *m *≤ *n *can be imposed with all generality.

We want to describe the evolution with time of the transition probabilities. For that purpose, I will use the *m *× *n *matrix of transitions *Q*_*t *_such that,



Note that an evolutionary model of the form *Q*_*t *_= *Q*_0_*e*^*tR*^, like that used for evolving gaps as extra characters, is not sufficient. A model of the form *Q*_*t *_= *Q*_0_*e*^*tR *^in the limit of infinite divergence would necessarily result in transitions that for a given end state are all identical and independent of the previous state. That is clearly too restrictive for most models, for instance in a profile HMM, in which some of the transitions are not evolved and are set to zero.

In order to allow for more general saturation properties of the transition probabilities, I propose the following model for the evolution of the matrix of transition probabilities,

*Q*_*t *_= *Q*_0 _+ *K *(*e*^*tR *^- *I*),     (114)

where *R *is the *n *× *n *rate matrix, and the *m *× *n *matrix *K *is still to be determined. This extension (as in the vector model proposed before) corresponds to adding a constant term *A *= *Q*_0 _- *K*, and it is is the more general solution of a differential equation of the form  ∝ (*Q*_*t *_- *A*).

We will see that *K*(*e*^*tR *^- *I*) = (*Q*_0 _- *Q*_∞_)(*e*^*tR *^- *I*), thus

*Q*_*t *_= *Q*_0 _+ (*Q*_0 _- *Q*_∞_) (*e*^*tR *^- *I*_*n *× *n*_),     (115)

= *Q*_∞ _+ (*Q*_0 _- *Q*_∞_) *e*^*tR*^.     (116)

As in the previous case, a freedom provided by the additional constant-in-time term is that while the saturation behavior of *Q*_0_*e*^*tR *^is controlled by the saturation probabilities of *e*^*tR*^, the model given by equation (116) is independent of those saturation probabilities so that the probabilities at infinity can be set arbitrarily. That is, assuming that *ψ *is the *n *dimensional vector of saturation probabilities of *e*^*tR*^,





Notice that while *Q*_*t*_, *Q*_0_, *Q*_∞ _and *K *are *m *× *n *matrices operating in the *S *× *E *space, the matrices *R *and *e*^*tR *^are square *n *× *n *matrices operating in the *E *× *E *space. In fact, *e*^*tR *^determines the change in time that a transition probability into one of the *E *states experiences and in which fashion that change is absorbed by the transition probabilities into any other *E *state.

##### A step-by-step description of the algorithm

The recipe to implement the algorithm is as follows:

1. Assume we know the *m *× *n *(*m *≤ *n*) matrices of transition probabilities at time zero *Q*_0 _and at time infinity *Q*_∞_, such that the rank of *Q*_0 _- *Q*_∞ _is m.

2. If an analytic *n *× *n *rate matrix *R *is given, one can find the analytic expression for *e*^*tR *^by solving the differential equation *d*(*e*^*tR*^)/*dt *= *Re*^*tR*^, and jump to step (6). For a numerical solution jump to step (5).

3. If the information given is the set of transition probabilities at a generating time t_*_, calculate the rate matrix *R *as,



The *n *× *m *matrix *O *is obtained by solving the set of linear equations

-(*Q*_∞ _- *Q*_0_) *O *+ *u*_*m*_*v*^*T *^= *I*_*m *× *m*_,     (120)

where *u*_*m *_is the *m *dimensional unity vector [*i.e. *], and *v *is a *m *dimensional vector uniquely determined by the set of *m *independent linear equations,

*v*^*T *^(*Q*_∞ _- *Q*_0_) = 0,     (121)

*v*^*T *^*u*_*m *_= 1.     (122)

The solution of equation (120) is not unique. In fact, equation (120) determines the matrix *O *up to a *n *dimensional probability vector *ψ *that satisfies the conditions *ψ*^*T *^*O *= 0. This probability vector corresponds to the saturation probabilities of the matrix *e*^*tR*^. While the rate matrix *R *and the matrix *e*^*tR *^depend on the choice of the saturation probabilities *ψ*, the asymptotic behaviour of the matrix of transition probabilities is independent of *ψ*, as was shown in equation (118).

4. Impose the condition,

*v*^*T *^(Q_* _- *Q*_0_) = 0.     (123)

This condition [necessary so that *ψ*^*T *^*R *= 0] imposes constraints between the set of probabilities at time zero, at time t_*_, and at time infinity.

5. Calculate the exponential of the rate matrix *e*^*tR *^using the corresponding Taylor expansion.

6. Finally, calculate the set of evolved transition probabilities as,

*Q*_*t *_= *Q*_0 _- (*Q*_∞ _- *Q*_0_)(*e*^*tR *^- *I*_*n *× *n*_)     (124)

or

*Q*_*t *_= *Q*_∞ _+ (*Q*_0 _- *Q*_∞_) *e*^*tR*^.     (125)

##### An example: Evolution of the transitions of a profile HMM given a rate matrix

To illustrate this method, consider the case of a profile HMM (Figure 4). There are three states associated with a given consensus position in the profile: the Match state (*M*), the Insert state (*I*) and the Delete state (*D*). These three states transition into states *M' *(the Match state at the next position in the profile), *D' *(the delete state at the next position in the profile), and *I *(the insert state between the two matched positions), therefore in this example *m *= 3 and *n *= 3.

Consider the following the transition matrix



This transition matrix, like a nucleotide substitution matrix, adds up to one by rows. We assume as in HMMER [4] that there are no transitions between the Insert and the Delete states, but the model could work under more general conditions.

The matrix at time zero is given by



The parameter 1/(1 - *q*_*I*_) is the average length of an insert in between two matched positions at very short times (if there were no deletions). The parameter 1/(1 - *q*_*D*_) is the average length of a deletion at very short times (if there were no insertions, and all position in the profile had the same parameters at time zero). For instance, one could set *q*_*I *_very close to zero, which implies that, for very small times when , the average length of a insertion would be very close to one.

At time infinity, one can parameterize the transition probabilities as



where *m*_*D *_and *m*_*D *_represent the probabilities of Match to Delete and Match to Insert at infinity, and *d*_*D *_and *i*_*I *_are the Delete to Delete and Insert to Insert probabilities at time infinity, (0 ≤ *m*_*D*_, *m*_*I*_, *d*_*D*_, *i*_*I *_≤ 1).

Let us assume that the rate matrix is given by



for some parameter *α *> 0. This rate matrix assumes that the rate of change in the occurrence of state *M' *is similar to that of state *D' *and that of state *I*, and that this change reverts equally into the other two states. More realistic situations can be achieved using rate matrices depending on more parameters.

The instantaneous rate of transition change is given by,



This matrix gives the instantaneous change that a transition probability experiences under this model and describes how that change is transferred to the other allowed transition probabilities.

The matrix *e*^*tR *^can be obtained analytically by solving the differential equation *d*(*e*^*tR*^)/*dt *= *Re*^*tR*^. This is a 3-dimensional Jukes-Cantor model which has as its solution,



with





Putting all together, we obtain the following evolved transition probabilities for a profile HMM under a Jukes-Cantor like assumption for the rate matrix:



Substituting the values for *s*_*t *_given in equation (133), we have the following evolved transition probabilities for a profile HMM,















##### The evolution of different paths through the HMM

In a tree-HMM one assumes that the different paths through the model are the objects that are subject to evolution [34]. Here we have directly modeled the evolution of the transition probabilities of the HMM. We can get an intuition for the meaning of these evolved transition probabilities by estimating how these evolved transition probabilities induce the evolution of different paths through the model. A process that is similarly to that modeled by a one-branch tree-HMM.

Suppose that at time zero, we emitted residue *a *from state *M*, and residue *b *from state *M'*. The model assigns to such sequence a probability given by,



where *p*_*M*_(*a*) and *p*_*M'*_(*b*) represent the emission probabilities associated to the *M *and *M' *states respectively. Now suppose that at time *t *there has been an insertion of *n *residues in between the two matches *a *and *b*; the model assigns to such sequence a probability given by,



where *p*_*I*_(*i*_*i*_) represent the emission probabilities associated to the Insert state.

We can interpret that in time *t *the path through the model that generated *ab *has evolved into the path through the model that generated *ai*_1_...*i*_*n*_*b *with probability given by



To get a better intuition of what this means, take as an example the case in which the time interval *t *is very small.

Then the probability that a path between two matches in the HMM inserts *n *residues in time *t *≈ 0 is



This probability is proportional to 3*αm*_*I *_the rate of substituting a Match-to-Match transition for a Match-to-Insert transition, and to  (1 - *q*_*I*_), which is the geometric factor associated to an insert of length *n *at time zero.

##### An example: Evolution of the transitions of a profile HMM given the transitions at a generating time

In this case, we maintain the same values for the transition probabilities at time zero *Q*_0 _and at time infinity *Q*_∞_, but the rate matrix will be obtained from a generating time for which we know the transition probabilities.

The set of linear equations in step (3) of this algorithm that determine the vector *v*^*T *^= (*v*_1_, *v*_2_, *v*_3_) are

*m*_*D*_*v*_1 _+ (*d*_*D *_- *q*_*D*_)*v*_2 _= 0,     (146)

*m*_*I*_*v*_1 _+ (*i*_*I *_- *q*_*I*_)*v*_3 _= 0,     (147)

*v*_1 _+ *v*_2 _+ *v*_3 _= 1.     (148)

The solution of these linear equations is

*v*_1 _= (*d*_*D *_- *q*_*D*_)(*i*_*I *_- *q*_*I*_)/*d*,     (149)

*v*_2 _= -*m*_*D*_(*i*_*I *_- *q*_*I*_)/*d*,     (150)

*v*_3 _= -*m*_*I *_(*d*_*D *_- *q*_*D*_)/*d*,     (151)

where *d *≡ (*d*_*D *_- *q*_*D*_)(*i*_*I *_- *q*_*I*_) - *m*_*D*_(*i*_*I *_- *q*_*I*_) - *m*_*I*_(*d*_*D *_- *q*_*D*_).

Parameterize the matrix *O *in the form



with each row adding to zero. The set of linear equations in step (3) that determine the matrix *O *are

(*d*_*D *_- *q*_*D*_)(*M*_1 _- *D*_1_) + *v*_1 _= 0, (*i*_*I *_- *q*_*I*_)(*M*_1 _- *I*_1_) + *v*_1 _= 0,     (153)

(*d*_*D *_- *q*_*D*_)(*M*_2 _- *D*_2_) + *v*_2 _= 1, (*i*_*I *_- *q*_*I*_)(*M*_2 _- *I*_2_) + *v*_2 _= 0,     (154)

(*d*_*D *_- *q*_*D*_)(*M*_3 _- *D*_3_) + *v*_3 _= 0, (*i*_*I *_- *q*_*I*_)(*M*_3 _- *I*_3_) + *v*_3 _= 1.     (155)

Solving *M*_*i *_and *I*_*i *_in terms of *D*_*i *_we have,



The matrix *O *is therefore determined up to the unitary vector (*D*_1_,*D*_2_, *D*_3_). The saturation probabilities *ψ*^*T *^= (*ψ*_*M*'_, *ψ*_*D*'_, *ψ*_*I*'_)(*ψ*^*T*^*R *= 0) are defined by the equations *ψ*^*T*^*O *= 0, which imply







Substituting vector *D *with vector *ψ *we finally obtain the following expression for the matrix *O *in terms of the saturation probabilities *ψ*:



The condition in step (4) of the algorithm translates in this case into the following relationship of parameters,



This is an additional set of constraints that the "vector" algorithm does not impose.

To test the algorithm I have made up a toy HMM consensus state, which at the generating time t_* _is given by the matrix of transitions,



Selecting the particular values *q*_*D *_= *q*_*I *_= 0.1 and *d*_*D *_= *i*_*I *_= 0.6, using the constraints of equations (161) implies that *m*_*D *_= 0.33 and *m*_*I *_= 0.25. Using these values and the arbitrary values for the saturation probabilities *ψ *= (1/3, 1/3, 1/3), we obtain the following *O *matrix:



The rate matrix *R *constructed using equation (119) is given by



and an instantaneous rate matrix -(*Q*_∞ _- *Q*_0_)*R *is given by,



The evolved set transition probabilities at time *t *= 0.3 is given by,



Using the "vector" method, in which transition probability vectors evolve independently with the same set of parameters, we would have obtained the identical result,



The normalization function *w*_*t *_given in equation (82) is different from zero only for dimensions larger than two. The second and third row effectively have dimension two (since one of the elements is always zero), and do not require normalization. For the first row the normalization function takes the value *w*_0.3 _= 0.0020.

The vector method allows us to use more unrestricted sets of parameters than the matrix method since the conditions in equation (78) are independent for each row. In principle, however, the conditions in equation (161) seem to allow behaviors that the vector model does not allow such as  >*m*_*D *_≡  as long as, simultaneously,  >*d*_*D *_≡ . In practice when I have tested that kind of situation, the rate matrices obtained are always not real, and therefore they lack any biological interpretation.

##### Derivation of the algorithm to evolve a matrix of transition probabilities

We start with a model of the general form

*Q*_*t *_= *Q*_0 _+ *K *(*e*^*tR *^- *I*_*n*×*n*_),     (168)

where *Q*_0 _is the known *m *× *n *matrix of probabilities at time zero, and the *m *× *n *matrix *K *must still be determined.

Assume that we know the transition probabilities at time infinity, which we represent by the *m *× *n *matrix *Q*_∞_. Then, because of the asymptotic behavior of the exponential family *e*^*tR*^, lim_*t*→∞_*e*^*tR *^= *u*_*n*_*ψ*^*T *^for some *n *dimensional saturation probabilities, where *ψ*^*T *^= (*ψ*_*i*_,...,*ψ*_*n*_), and the *n *dimensional unity vector  = (1, ..., 1), we have

*Q*_∞ _= *Q*_0 _+ *K *(*u*_*n*_*ψ*^*T *^- *I*_*n*×*n*_).     (169)

This equation implies that

*K *= -(*Q*_∞ _- *Q*_0_)+ *ψ*^*T*^,     (170)

where  is a *m *dimensional vector that represents the sums by rows of *K*, *i.e. *∑_*j*_*K*(*i*, *j*) =  which we impose to be different from zero.

Because for the exponential family *e*^*tR *^we have the reversibility condition *ψ*^*T*^*e*^*tR *^= *ψ*^*T *^for arbitrary time, introducing the expression for *K *in equation (170) in the equation (114) we have the general result,

*Q*_*t *_= *Q*_0 _- (*Q*_∞ _- *Q*_0_)(*e*^*tR *^- *I*_*n*×*n*_).     (171)

This result proves point (6) of the previous algorithm description.

Therefore if given *Q*_0_, *Q*_∞ _and a *n *× *n *rate matrix *R*, which satisfy the reversibility conditions *ψ*^*T*^*R *= 0, we can calculate the evolved transition probabilities using the equation (171).

In the case in which the information given is the set of transition probabilities at a generating time t_*_, designated by Q_*_, the calculation of the rate matrix *R *involves the following steps:

**(a) **The *m *× *n *matrix *K *is by construction invertible because we have imposed  ≠ 0, for all rows *i*.

A little aside with respect to matrix inversions is in order here. The (unique) inverse of a matrix is defined only for square matrices. One can introduce a inverse-like matrix for a non-square matrix; these are called pseudoinverses [69]. The pseudoinverse of a non-square matrix is not unique and many pseudoinverses can be defined; one of the best known is the Moore-Penrose matrix inverse [70]. We will see how despite the fact that the pseudoinverse of *K *is not unique, we can still define *Q*_*t *_uniquely.

Therefore solving for *R *in equation (114) at the particular time t_* _we have



where *K*^-1 ^is the *n *× *m *pseudoinverse of *K *defined by the conditions *KK*^-1 ^= *I*_*m*×*m *_and *K*^-1^*K *= *I*_*n*×*n*_.

**(b) **Because the final result for *Q*_*t *_in equation (116) does not depend on the values  we can set them with all generality to the form  = *ρ *≠ 0. Therefore we have

*K *= -(*Q*_∞ _- *Q*_0_) + *ρu*_*m *_*ψ*^*T*^.     (173)

Because *K*^-1^*Ku*_*n *_= *u*_*n *_and *Ku*_*n *_= *ρu*_*m*_, then we need that *K*^-1^*u*_*m *_= *ρ*^-1^*u*_*n*_. Therefore we propose that the *n *× *m *pseudoinverse matrix *K*^-1 ^has the following form,



where the *n *× *m *matrix *O*, and the *m *dimensional vector *v *satisfy the conditions,

*O u*_*m *_= 0,     (175)

*v*^*T*^*u*_*m *_= 1.     (176)

**(c) **In order to satisfy *K*^-1^*K *= I_*n*×*n *_we need to have,

*v*^*T*^(*Q*_∞ _- *Q*_0_) = 0,     (177)

*-O*(*Q*_∞ _- *Q*_0_) + *u*_*n*_*ψ*^*T *^= *I*_*n*×*n*_.     (178)

Equation (177) is a set of homogeneous linear equations that together with the normalization conditions in equation (176) uniquely determine the vector *v*.

On the other hand, in order to satisfy *KK*^-1 ^= *I*_*m*×*m*_, the following must apply:

*ψ*^*T*^*O *= 0,     (179)

-(*Q*_∞ _- *Q*_0_)*O *+ *u*_*m*_*v*^*T *^= *I*_*m*×*m*_.     (180)

Equation (180) is a set of linear equations which determines *O *aside from a dependence on an arbitrary probability vector. In particular we can find the expression of matrix *O *in terms of the vector *ψ *as we did in equation (160).

Once the matrix *O *has been obtained using equation (180) as a function of the vector *ψ*, one can verify that the set of equations describe by (178) is automatically satisfied for any vector *ψ *as long as it satisfies the condition *ψ*^*T*^*O *= 0. This is the result presented in step (4) of the algorithm.

**(d) **Because *ψ *corresponds to the saturation probabilities of *e*^*tR*^, then it is necessary that *ψ*^*T*^*R *= 0. This condition is satisfied if,



Therefore it implies that,

*v*^*T*^(Q_* _- *Q*_0_) = 0,     (182)

which is the condition imposed in step (4) of the algorithm. Under those conditions, it results for the rate matrix *R*,



Notice that the parameter *ρ *≠ 0, which is necessary to be able to invert the matrix *K *to calculate the rate *R*, does not appear anywhere in the final result, either in the evolved transitions *Q*_*t *_or in the value of *R*. This results from the fact that in either equation the only relevant component is the projection of *K *(or *K*^-1^) into (*e*^*tR *^- *I*). The same projection is what makes the vector *ψ *that appears in the pseudoinverse *K*^-1 ^irrelevant. Even though lim_*t*→∞_*e*^*tR *^= *uψ*^*T*^, it is also true that lim_*t*→∞ _(*Q*_∞ _- *Q*_0_)*e*^*tR *^= 0, so that the dependence on *ψ *disappears from the final expression of *Q*_∞_.

### Reversibility and multiplicativity

For a given probabilistic model, imposing reversibility has different implications for its emission and transition probabilities. In pair models, we assume that the emission probabilities are reversible by imposing *P*(*a*_*t*_, *b*_*t*+*t'*_) = *P*(*a*_*t*+*t'*_,*b*_*t*_), which corresponds to using symmetric joint probabilities represented by the shorthand notation *P*(*a*, *b*|*t'*). If the emissions do not involve gaps, the marginal probabilities do not evolve, and the evolved joint probabilities are obtained from the evolved conditionals and the saturation probabilities. In the presence of gaps, I have described how to construct the evolved conditionals and the corresponding evolved marginals in a way that maintains reversibility for any arbitrary time, so that we can construct evolved symmetric joint probabilities.

For transition probabilities the situation is different. Mathematically, a matrix of transition probabilities is like a substitution matrix (*i.e*. conditional probabilities) but there is not the equivalent of "joint" probabilities for transitions. To maintain reversibility for the transitions of a probabilistic model, one has to build reversibility in the design of the model. In particular, one needs to be sure that the transition probabilities that involve gaps lack any directionality. For instance, in the pair-HMM of Figure 3 we need to impose that  for arbitrary times. That is achieved by making sure that the input transition probabilities at time t_*_, zero and infinity do lack directionality.

Another property of probabilistic models of evolution for residue substitutions is multiplicativity. Multiplicativity is an immediate property for evolutionary models of the form *e*^*tR*^. For residue-substitution evolutionary processes, multiplicativity implies that the transition from one given event (say residue *a*) to another event (say residue *b*) in a finite time, if it goes through any intermediate state, has to be of the form of any other possible substitution. In mathematical terms,



However, when allowing gaps, any intermediate evolutionary step can go through processes of deletions or insertions in addition to substitutions; therefore multiplicativity as described in the previous equation does not hold anymore. There is a natural explanation of why "substitutions-only multiplicativity" is modified when considering insertion and deletion events. Consider the evolution of gaps as single characters, which was introduced previously in this paper. The substitution matrix with gaps  satisfies the relationship



Analyzing this matrix equation by components and using the expression for *Q*_0 _given in equation (22), the substitution of residue *a *into residue *b *in finite time *t *+ *t' *has the following terms:



The first term corresponds to pure substitution events of the form , and it is identical to equation (184). The second term modulated by the coefficient 1/*q*_0 _(introduced in equation (65), which is part of the non trivial matrix *Q*_0_) represents the event in which . The third term (preceded by coefficient (1 - 1/*q*_0_)) represents the event in which . Note that this model would align at time *t *+ *t' *residues which could have been derived by a gap intermediate. This is usually discouraged by evolutionary models that describe the evolutionary history of insertions and deletions, in which such event would be represented as . For the model at hand, the fact that a gap can revert into a residue is a consequence of treating gaps as an additional residue in a substitution matrix.

For the particular case of the generalized Jukes-Cantor model introduced before, it turns out that the two extra terms in equation (186) are independent of the particular substitutions and cancel, such that



Therefore the generalized Jukes-Cantor model preserves multiplicativity. This results from the extreme simplicity of the model and is not true for more complicated models. For instance, for the rate matrix created from a particular Q_* _in the other example presented in this paper (which is a particular case of the REV model [44]), the two extra terms in equation (186) are different for the different nucleotide substitutions, and do not cancel out.

A more complicated situation appears for probabilistic models that introduce gaps in an affine manner. A given residue-to-residue substitution process that occurred in a finite time could have appeared from a very large number of intermediate situations in which stretches of other nucleotides could have been added or removed. The simple one-to-one correspondence that models of substitutions maintain through evolution does not exist in the presence of insertion and deletion events. This does not mean that evolutionary models with gaps are inconsistent, however some traditional properties of phylogenetic trees of single residue evolution such as the pulley principle [71] cannot be applied under the transition probability evolution models.

## Conclusion

Motivated by the goal of making QRNA (a comparative probabilistic method for RNA genefinding) an evolutionary model, I have introduced several probabilistic methods to describe the evolution of insertion and deletion events. The methods introduced here have a larger scope than this program alone, and they can be applied to other pair probabilistic models and to profile HMMs and SCFGs as well.

I described an algorithm which addresses the evolution of gaps as an extra residue in a (*N *+ 1) × (*N *+ 1) substitution matrix. This method can be applied to the joint emission probabilities of pair models. This method allows us to maintain a stationary *N*-dimensional background distribution, while the actual (*N *+ 1)-dimensional background frequencies evolve towards all gaps at time infinity. I call this process quasi-stationary. As an example, I showed an analytic solution for the Jukes-Cantor model extended to gaps.

I also presented two methods for the evolution of transition probabilities in a profile or pair HMM or SCFG, that are applicable to any probabilistic model that uses transitions between states to model insertions and deletions. In the first algorithm, the transition probabilities associated with one state in the model are evolved as a vector independently of the transition probabilities associated to any other state in the model. I also presented a second algorithm in which the transition probabilities associated with a given set of states co-evolve under the control of a single rate matrix. I presented an example of the application of these methods to a pair-HMM and to a profile HMM.

I have applied these methods to the program QRNA, which was the motivation for the development of the algorithms in the first place. QRNA contains three probabilistic models (the oth, cod, and rna models) that analyze the pattern of mutation of a given pairwise alignment to decide which of the three models best classifies the alignment. These models are a combination of generalized pair-HMMs and a pair-SCFG. Originally, this program assumed a fixed divergence time, and all the emission probabilities of the different models were tied to those of BLOSUM62. That produced a QRNA parameterized for highly diverse sequences, which in turn produced a large number of false positives for highly similar sequences. In the new program eQRNA, all emission and transition probabilities are a continuous time-dependent family able to match any possible degree of sequence divergence.

The three models of QRNA (the OTH, COD, and RNA models) need to be at approximately the same evolutionary distance, so that when a pairwise alignment is analyzed, the differences in scores of the models result from observing a different pattern of mutations (coding, RNA, or none in particular) rather than because one model favors more closely related sequences than the other. This model synchronization requires a number of QRNA-specific design elements which are tangential to the implementation of the evolutionary models for indels and transition probabilities presented in this paper. For reasons of clarity, I leave for another paper a detailed description of the particular implementation designs that went into eQRNA in order to make it fully evolutionary. In a nutshell, the transition probabilities of the OTH and COD models are evolved according to the algorithm to evolve vectors of transition probabilities, while the emission probabilities of those two models were evolved using the original QRNA parameters as the generating time of the respective rate matrix. In the RNA model, for the context-free grammar component of the model, the transitions are fixed, and the evolution of gaps is accommodated by treating gaps as extra characters according to the method presented here for that purpose. The HMM component of the RNA model is parameterized with time similarly to the OTH and COD models. Preliminary results show an important improvement compared with the previous fixed-time implementation. The application of these evolutionary methods for other probabilistic models for sequence comparison beyond eQRNA should be tractable.

So far the methods presented here have been introduced only in profile and pair models. They could also be applied to probabilistic models where, instead of aligning two contemporary sequences, one aligns a sequence to an ancestor. The only difference with respect to an evolutionary pair model is that, in this case, the emission probabilities will be the substitution (conditional) matrices themselves instead of joint conditional-on-time probabilities. One important limitation of the methods presented here is that, in general, they lack the property of multiplicativity. In consequence, in order to extend the methods presented here to more than two sequences related by a phylogenetic tree, one would have to work with rooted trees. A future challenge is to incorporate these evolutionary methods into multiple sequence probabilistic models that explicitly describe the phylogenetic relationship between the sequences.

## Availability

The different models presented in this paper have been implemented in several small ANSI C programs. These are not fully developed software applications, but demonstrations (for those who want to avoid the mathematical descriptions) of how the different algorithms work. The programs are freely available at .

## Methods

### Appendix A. Conditions for the saturation of a generalized substitution matrix

In this appendix I provide the conditions for saturation of a generalized evolutionary model of the form *Q*_*t *_= *Q*_0_*e*^*tR*^. Saturation can be described as



for the unitary vector *u*, and a set of saturation frequencies at time infinity denoted by *q*_∞_, such that .

Here I show that saturation of *Q*_*t *_= *Q*_0_*e*^*tR *^is a necessary condition of two properties of the matrix *Q *= {*Q*(*ij*)}, normalization and positivity. I also show that the saturation probabilities of *Q*_*t *_are the same as those of *e*^*tR*^.

**Proposition A.1. **Consider first the simplest case *Q*_*t *_= *e*^*tR*^. Normalization, *i.e*. ∑_*j*_*Q*(*ij*) = 1, together with positivity, *i.e*. *Q*(*ij*) > 0 ∀*i*, *j*, imply that a substitution matrix of the form *Q*_*t *_= *e*^*tR *^saturates to a set of probabilities at time infinity.

*Proof*. Normalization of the rate matrix, ∑_*j *_*Q*(*ij*) = 1 implies that



That is, *λ *= 1 is an eigenvalue of *Q*. It also has implications for the norm of *Q*, defined as the largest row sum of absolute values



Therefore, because of the spectral theorem [72], the spectral radius *σ*(*Q*), defined as the largest absolute value of any eigenvalue of *Q*, is bounded by,

*σ*(*Q*) ≤ ||*Q*|| = 1.     (191)

On the other hand there is an eigenvalue *λ *= 1 therefore

*σ*(*Q*) = 1.     (192)

In consequence, *Q *has one eigenvalue, *λ *= 1, and all other eigenvalues are smaller than one.

Therefore because the substitution matrix is of the form *Q*_*t *_= *e*^*tR*^, it implies that the instantaneous rate matrix *R *has one null eigenvalue, and all the other are negative. If we assume that the null eigenvalue is not degenerate and that the negative eigenvalues are real, we can write with all generality,



for some matrix *U*, and such that *λ*_*i *_> 0 for *i *= 2, ..., *n*.

Therefore *Q*_*t *_= *e*^*tR *^can be cast into the form,



In the limit,



for  = (1, 0, ..., 0).

On the other hand using equation (194) we obtain



which implies that *U*Ψ_0 _is the eigenvector of *Q *corresponding to the eigenvalue *λ *= 1. According to (189) that is,



Substituting in equation (195) we finally obtain,



This is the saturation condition (188) for some saturation probabilities defined by *q*_∞ _= *U*^-1^.

**Corollary A.1. **For a generalized evolutionary model of the form *Q*_*t *_= *Q*_0_*e*^*tR*^, *Q*_*t *_also saturates at infinity, and the saturation probabilities of *Q*_*t *_are given by those of *e*^*tR*^, that is,



*Proof*. Note that by construction *Q*_0 _has to have the same normalization and positivity conditions as *Q*_*t*_. It can be shown that under those conditions, *Q*_*t *_= *e*^*tR *^also has to add up to one, summing by rows, and all its elements have to be positive. Therefore, using the result of Proposition A.1,



Therefore



which proves saturation for an evolutionary probabilistic process of the form *Q*_*t *_= *Q*_0 _*e*^*tR*^.

### Appendix B. Implications of reversibility on a generalized evolutionary process

In this appendix I discuss the implications that reversibility imposes on a generalized evolutionary model. I show that for an evolutionary model of the form *Q*_*t *_= *e*^*tR*^, the marginal probabilities with respect to which *Q*_*t *_is reversible have to be stationary, and therefore coincide with the saturation probabilities. I also show that for an evolutionary model of the general form *Q*_*t *_= *Q*_0_*e*^*tR*^, the marginal probabilities with respect to which *Q*_*t *_is reversible can change with time. In this way we decouple the "reversibility" frequencies from the saturation frequencies. I also demonstrate how to calculate the saturation probabilities, given *Q*_0 _and Q_* _at one particular time t_*_. This system sets the ground for the quasi-stationary model of evolution with gaps as an extra indel.

**Lemma B.1. **Consider a given matrix of conditional probabilities Q_*_, [∑_*j *_Q_*_(*ij*) = 1 ∀*i*] which is reversible with respect to a set of marginal probabilities p_*_,

p_*_(*i*)Q_*_(*ij*) = p_*_(*j*)Q_*_(*ji*).     (202)

Then one can see that reversibility implies



*Proof*. Summing one of the indices in the reversibility conditions and taking into account the normalization condition for the Q_* _matrix results in,



which in vectorial notation takes the form 

**Lemma B.2. **If *R *= log Q_* _then the reversibility condition (202) for Q_* _implies that



*Proof*. If *R *= log Q_* _then because of the Taylor series we have



Because of the reversibility condition for Q_* _(202) it is also true that

p_*_(*i*) (Q_* _- *I*)^*n *^(*ij*) = p_*_(*j*)(Q_* _- *I*)^*n *^(*ji*),     (207)

for *n *≥ 1. Therefore it follows that

p_*_(*i*) *R*(*ij*) = p_*_(*j*)*R*(*ji*).     (208)

In addition we can also see by inspecting equation (206) that the normalization condition for Q_* _translates into ∑_*j *_*R*(*ij*) = 0 ∀*i*, which implies that



**Lemma B.3. **If Q_* _is a conditional matrix that satisfies the reversibility condition (202) and *R *= log Q_* _then the saturation probabilities of *R *are given by the p_* _vector in (202), that is,



*Proof*. Taking from *Lemma B.2.*, we have ; therefore  for *n *≥ 0, and because of the relationship



it results that



for arbitrary t. Therefore, it also holds in the limit of very large time that



Additionally, Appendix A shows that . Combining those two equations together we have



This proves that the saturation probabilities are p_*_.

**Proposition B.1. **For a reversible evolutionary model of the form *Q*_*t *_= *e*^*tR*^, it results that the associated marginal probabilities with respect to which the parametric family *Q*_*t *_is reversible have to be stationary (*i.e*. time independent).

*Proof*. From the parametric family *Q*_*t *_select one particular instance t_*_, and consider . Suppose that the marginal probabilities at this time are given by p_*_, that is: . Because of the relationship *R *= log Q_*_, it follows from Lemma B.3 that the whole parametric family *e*^*tR *^has p_* _as the corresponding marginal probabilities, therefore the marginal probabilities do not evolve with time (stationary).

**Proposition B.2. **For a reversible generalized evolutionary model of the form *Q*_*t *_= *Q*_0_*e*^*tR*^, the associated marginal probabilities with respect of which *Q*_*t *_is reversible can be evolved with time.

*proof*. In order to prove that this is the case, we just need to find an example in which that statement is true. Consider again one particular instance  with its corresponding marginal probabilities p_*_. Because the model is reversible for arbitrary divergence times, in particular there should be some *p*_0 _probabilities such that . For this generalized model, the rate matrix is given by . Therefore it follow by Lemma B.3 that the saturation probabilities of *R *are given by the condition



Therefore the saturation probabilities *p*_∞ _are different from p_* _as long as *p*_0 _≠ p_*_.

Therefore, we have constructed a parametric family, *Q*_*t *_= *Q*_0_*e*^*tR*^, in which the marginal probabilities for reversibility are *p*_0 _at time zero, p_* _at t_*_, and *p*_∞ _at time infinity, with *p*_0 _≠ p_* _≠ *p*_∞_. Therefore if there is reversibility at arbitrary time, the marginals have to be time dependent,

*p*_*t *_(*i*)*Q*_*t*_(*ij*) = *p*_*t*_(*j*)*Q*_*t *_(*ji*).     (216)

In particular in the Section "The evolution of emission probabilities with indels treated as an extra character" we have constructed a system in which the time-dependent reversibility condition (216) is satisfied by marginal probabilities that are quasi-stationary with respect to some (*n *- 1) *p*_0 _probabilities,

*p*_*t*_(*i*) = *p*_0_(*i*) (1 - Λ_*t*_), for *i *= 1,...*n *- 1     (217)

*p*_*t*_(*n*) = *Λ*_*t*_.     (218)
